# Fusing Multi-Temporal Context for Image Super-Resolution Reconstruction in Cultural Heritage Monitoring

**DOI:** 10.3390/s26010228

**Published:** 2025-12-30

**Authors:** Caiyan Chen, Fulong Chen, Sheng Gao, Hongqiang Li, Xinru Zhang, Yanni Cheng

**Affiliations:** 1 Key Laboratory of Digital Earth Science, Aerospace Information Research Institute, Chinese Academy of Sciences, Beijing 100094, China; chencaiyan19@mails.ucas.ac.cn (C.C.);; 2 International Research Center of Big Data for Sustainable Development Goals, Beijing 100094, China; 3 University of Chinese Academy of Sciences, Beijing 100049, China; 4 Kashi Key Lab of Big Earth Data and Sustainable Development Goal, Kashi Aerospace Information Research Institute, Kashi 844000, China

**Keywords:** remote sensing, deep learning, super-resolution

## Abstract

**Highlights:**

**What are the main findings?**

- A novel multi-branch, temporal change-aware super-resolution model is proposed, which explicitly leverages landscape evolution patterns from adjacent years to reconstruct high-quality imagery for a target year with missing data.
- The proposed model significantly outperforms both single-image super-resolution baselines and a multi-temporal ablation model, achieving superior results on a real-world heritage dataset of the Weiyang Palace site.

**What are the implications of the main findings?**

- This study transforms super-resolution from a simple image enhancement tool into a data-repair engine for dynamic monitoring, generating reconstructions that are consistent with the site’s temporal evolution logic.
- The method provides a practical solution to the “temporal data gap” problem in heritage monitoring, establishing a reliable data foundation for high-precision tasks like change detection and long-term preservation planning.

**Abstract:**

Effective conservation of World Heritage Sites relies on high-precision and continuous dynamic monitoring of their status. However, cloud cover, limitations in sensor resolution, and the vast distribution of heritage areas make it challenging to consistently acquire high-resolution imagery for key years, thereby hindering accurate characterization of their temporal evolution. To overcome this bottleneck, this paper proposes a temporal change-aware super-resolution reconstruction model. This model innovatively utilizes the temporal evolution information of heritage landscapes as a key clue for reconstructing high-quality imagery of the target year. We design a multi-branch architecture that takes the low-resolution image of the target year as the core input, while also incorporating the high- and low-resolution images from its preceding (t − 1) and subsequent (t + 1) years. Through parallel encoding branches, the model separately learns to: (1) extract spatial features from the multi-temporal low-resolution images, and (2) explicitly model the change patterns recorded in the high-resolution imagery from year t − 1 to t + 1, via a dedicated temporal change encoder. Finally, by deeply fusing these features, the model generates a simulated high-resolution image for the target year (t). Experimental results on a real-world dataset of the Weiyang Palace (WYP) core area (2017–2019), with 2018 as the target year, demonstrate that the proposed method achieves superior performance, significantly outperforming traditional single-image super-resolution models and a contrastive model without explicit temporal change modeling.

## 1. Introduction

As non-renewable cultural resources, the scientific and forward-looking nature of conservation and management practices for World Cultural Heritage sites is of paramount importance [1,2,3,4,5]. High-spatial-resolution remote sensing technology, with its macroscopic, objective, and periodic observation capabilities, has become an indispensable tool in various domains such as heritage mapping, damage identification, and environmental assessment [6,7,8,9,10,11,12]. In particular, by analyzing time-series imagery, managers can track the stability of heritage structures, changes in the surrounding ecological environment, and the impact of human activities, thereby enabling precise preventive conservation [13,14,15,16,17].

However, a long-standing technical contradiction hinders the effectiveness of temporal analysis: the need for fine-grained monitoring requires imagery with high spatial resolution at the meter or even sub-meter level [18,19,20,21]. Yet, due to the physical limitations of satellite sensors, high data acquisition costs, and unavoidable atmospheric interference (e.g., clouds, fog), obtaining a continuous, complete, and consistent high-resolution image sequence remains challenging [22,23,24,25,26,27,28]. This often results in situations where the only available data at critical monitoring time points are low-resolution images, creating “data gaps” in the dynamic monitoring chain of heritage sites.

Image super-resolution (SR) technology is one of the effective computational approaches to bridge this “data gap.” In recent years, deep learning-based single-image super-resolution has made significant progress, with models from SRCNN [29] to EDSR [30], RCAN [31] and beyond, continually advancing reconstruction capabilities [32,33,34,35,36,37]. Nevertheless, these mainstream methods share a common paradigm: they rely solely on information extracted from a single low-resolution image to reconstruct its high-resolution version [38,39,40,41,42,43]. This paradigm has inherent limitations when processing heritage time-series data: the reconstruction results lack temporal consistency [44,45]. In other words, the imagery reconstructed by traditional methods for a specific year may fail to accurately represent the true state of that year within the continuous evolution of the heritage site, as it completely ignores the landscape characteristics and change trends exhibited in the past and future.

In fact, image sequences of the same heritage area acquired over time contain rich temporal evolution logic. For instance, the protective restoration of a historic building might span several years, and the surrounding vegetation exhibits interannual cyclical changes. While these changes are the very objects of monitoring, their inherent continuity and regularity also provide strong physical constraints for reconstructing imagery of missing years. Recently, a few studies have begun to explore the use of multi-temporal images in SR [46,47,48,49,50]. For example, Caballero et al. introduce spatio-temporal sub-pixel convolution networks that effectively exploit temporal redundancies and improve reconstruction accuracy while maintaining real-time speed [46]. Meanwhile, Haris et al. integrate spatial and temporal contexts from continuous video frames using a recurrent encoder–decoder module that fuses multi-frame information with the more traditional, single frame super-resolution path for the target frame [47]. However, most focus on short-term motion compensation in video super-resolution and lack architectures specifically designed for the characteristics of remote sensing imagery—particularly heritage landscapes—which involve long time series and non-uniform change rates.

Based on this, our study aims to address a key problem in the dynamic monitoring of heritage sites: how to leverage the complete temporal context of an area (including its past and future landscape states) to reconstruct imagery for missing or poor-quality key years, ensuring the results are temporally plausible and consistent. We hypothesize that the change trajectory of a heritage site, as recorded in high-resolution images from adjacent years, provides crucial cues for inferring the image state of the target year.

We propose a temporal change-aware, multi-branch super-resolution reconstruction model. Building upon traditional image reconstruction, this architecture incorporates a novel temporal change learning branch to explicitly extract the evolving patterns of heritage landscapes. This enables deep collaboration of multi-temporal, multi-resolution remote sensing information, ultimately generating super-resolution results that exhibit both high spatial detail and temporal consistency. We conducted systematic experiments on a real remote sensing dataset from the Weiyang Palace (WYP) Heritage Site in Shaanxi Province. The results demonstrate that our method not only improves quantitative metrics but also shows significant advantages in detail fidelity and temporal consistency for typical heritage features (e.g., ancient building complexes, historical landscapes), providing more reliable data support for dynamic monitoring of heritage sites.

## 2. Materials and Methods

### 2.1. Study Area and Data

**Figure 1:**
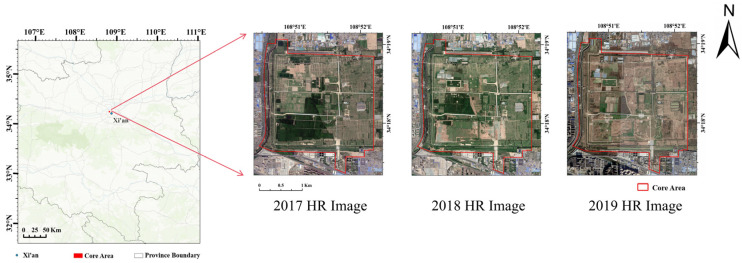
Location and high-resolution imagery of the study area.

This study selects the core area of the WYP as the research region (Figure 1). As a significant capital site dating to the Han period, this site possesses the following typical characteristics and research value: (1) WYP represents a crucial milestone in the evolution of the ancient Chinese capital system, where its city wall layout, palace foundations, and ritual structure remnants reflect the urban planning philosophy and social structure of that era. (2) The site area exhibits typical loess landforms, with surface cover including exposed earthen ruins, restored exhibition buildings, vegetated areas, and modern artificial features, providing a complex scene of ground objects for remote sensing image analysis. (3) Due to the vast scale and complex preservation status of the site, traditional ground-based monitoring methods struggle to achieve comprehensive coverage, creating a pressing need for efficient and continuous dynamic monitoring via remote sensing technology. The diverse land cover types and distinct spatiotemporal change patterns in this area make it highly suitable for validating the effectiveness of the proposed temporal-aware super-resolution method in cultural heritage monitoring.

#### 2.1.1. Data Source and Preprocessing

This study utilized remote sensing image data from three consecutive years (2017 to 2019), all sourced from the Google Earth platform. The selection of these consecutive years aimed to capture coherent temporal evolution patterns (e.g., multi-year restoration progress) while minimizing potential inconsistencies arising from drastic sensor changes or long-term land cover shifts. The dataset was constructed following this procedure:

Raw Data Acquisition: High-spatial-resolution imagery (spatial resolution: 0.5 m) for the three years was acquired to serve as the ground-truth reference data. Corresponding low-resolution image sets (spatial resolution: 4 m) were generated by downsampling the high-resolution imagery, simulating the common scenario of medium-to-low resolution data in practical monitoring. This controlled simulation approach inherently mitigates the impact of cross-sensor variability and acquisition condition differences (e.g., view angle, atmospheric effects) on the fusion process, allowing us to better evaluate the core contribution of the temporal fusion mechanism.

Geometric Registration: A feature-based registration algorithm was employed to strictly co-register all images to the same geographic coordinate system (WGS 1984 UTM Zone 49N), achieving sub-pixel alignment accuracy (RMSE < 0.5 pixels) for multi-temporal images and establishing a foundation for subsequent pixel-wise temporal analysis.

Radiometric Normalization: All images underwent radiometric correction and normalization to mitigate radiometric biases caused by variations in illumination conditions, sensor differences, and atmospheric conditions among different temporal images, ensuring comparability.

Region Cropping and Sample Generation: Following the aforementioned preprocessing steps (registration and normalization) applied to the full-scene images, the processed high-resolution images were cropped into 512 × 512 pixel patches using a regular grid with a stride of 256 pixels. These high-resolution patches were then used to generate their corresponding low-resolution counterparts, forming the sample pairs for model training and validation. These samples were randomly split into training, validation, and test sets in a 3:1:1 ratio.

#### 2.1.2. Experimental Data Configuration

To validate the capability of our method in reconstructing high-resolution imagery for a missing year, the following experimental setup was defined:

Target Year: The year 2018 was designated as the target year for reconstruction (denoted as t).

Input Data: The model inputs include: The low-resolution image of the target year t; The high-resolution and low-resolution image pairs from the preceding year (t − 1, i.e., 2017) and the subsequent year (t + 1, i.e., 2019).

Reconstruction Target: Using the aforementioned multi-temporal data, the goal is to reconstruct the high-resolution image (0.5 m) for the target year t.

This configuration simulates a common data gap scenario in cultural heritage monitoring and focuses on evaluating the performance of the proposed method in integrating multi-temporal contextual information to enhance reconstruction quality and temporal consistency.

### 2.2. Temporal Change-Aware Multi-Branch Model

**Figure 2:**
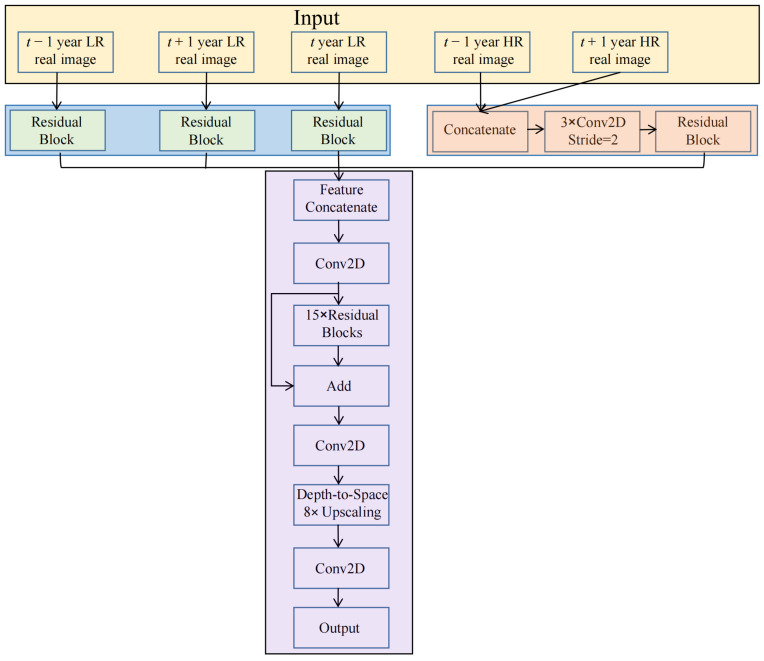
Overall architecture of the temporal change-aware multi-branch model.

The overall architecture of our proposed model is illustrated in Figure 2. It is a carefully designed five-input, single-output network, whose core idea is to leverage temporal change cues through multi-path information extraction to reconstruct high-resolution heritage imagery for the target year.

As shown in Figure 2, the model accepts five input sources: the low-resolution image of year t − 1 (X_lr_t − 1), the high-resolution image of year t − 1 (X_hr_t − 1), the low-resolution image of year t + 1 (X_lr_t + 1), the high-resolution image of year t + 1 (X_hr_t + 1), and the low-resolution image of the target year t (X_lr_t). These inputs are processed in parallel through several branches to extract feature information from different dimensions.

Multi-temporal Spatial Context Branch: This branch aims to extract robust, resolution-invariant spatial features from the temporally adjacent low-resolution images (X_lr_t − 1, X_lr_t + 1) and the target low-resolution image (X_lr_t). To achieve this, we employ a feature extraction module, built with a residual block, which is shared across these three inputs. The residual block contains two convolutional layers, with Batch Normalization and ReLU activation embedded between them to accelerate convergence and enhance non-linear representation capability. Furthermore, a scaling factor (set to 0.1) is introduced at the end of the residual path to stabilize the training of the deep network by scaling down the residual signal. This design enables the model to focus on the generic spatial structures in the imagery, ensuring a profound understanding of the fundamental layout and characteristics of the heritage area, thereby providing stable and reliable spatial context information for subsequent fusion and reconstruction modules.

Temporal Change Learning Branch: This branch represents the core innovation of our model, specifically designed to explicitly extract temporal evolution information of the heritage site from X_hr_t − 1 and X_hr_t + 1. This branch first concatenates the two high-resolution images from the adjacent years along the channel dimension. The concatenated input is then passed through a downsampling path composed of three strided convolutional layers (stride = 2, filter size 3 × 3, each with 48 output channels), progressively reducing the feature map size. The resulting features are fed into a residual block to extract temporal change characteristics. This process forces the network to learn a compact feature representation that encapsulates the key land surface change patterns occurring from year t − 1 to t + 1. This representation potentially encodes crucial temporal context for heritage monitoring, including which areas changed, the trend and magnitude of changes, and which core heritage elements remained stable. It thereby provides strong temporal constraints for accurately reconstructing the target year’s image.

Multi-temporal Fusion Mechanism: In the feature fusion stage, the output feature maps from the above-mentioned four branches are concatenated along the channel dimension to form a comprehensive fusion feature: F_fusion = Concat(F_t − 1, F_t + 1, F_temporal, F_t). This concatenation operation initially treats information from all temporal sources equally. However, the subsequent Refinement Module (detailed below) implicitly learns to weight and integrate these features. This fused feature incorporates not only direct observational information from the target year and its adjacent years but also integrates powerful constraints from the temporal dimension, laying a solid foundation for high-quality reconstruction. This design allows the model to adaptively emphasize more reliable or relevant temporal cues during fusion.

The fused features are subsequently fed into a Refinement Module, which consists of 15 consecutive residual blocks (each with 64 filters). This module facilitates the effective integration of multi-source feature information through deep non-linear transformations. Notably, it can organically associate temporal change cues with spatial structural features, ensuring that the reconstruction results maintain both spatial detail accuracy and temporal evolution logic. It is within this module that the model learns to handle potential inconsistencies or incomplete data across time points by relying on complementary information from other branches.

Finally, the refined features enter the upsampling reconstruction module. Our model employs efficient sub-pixel convolution for upsampling. This operation first expands the number of channels to the square of the target scale factor via a convolutional layer, then directly increases the spatial size of the feature maps through a pixel shuffling operation. Compared to traditional interpolation-based upsampling, this method better preserves detailed information. A final convolutional layer outputs the reconstructed result Y_hr_t. The overall design ensures that the model, while fully utilizing multi-temporal information, generates high-resolution heritage imagery that is more consistent in both spatial detail and temporal aspects.

### 2.3. Loss Function and Training Strategy

To guide the model in generating reconstruction results with high fidelity in both pixel accuracy and visual quality, while ensuring an efficient and stable training process, this study designs a composite loss function and adopts a refined end-to-end training strategy.

#### 2.3.1. Composite Loss Function

We employ a composite loss function, *L_total_*, to supervise the final reconstruction output of the model (i.e., the predicted high-resolution image for year t). This loss function consists of a combination of Mean Squared Error (MSE) loss and Structural Similarity Index Measure (SSIM) loss, expressed as:

$$L_{total}=\alpha \times L_{MSE}+{\beta \times L}_{SSIM}$$

where:

*α* and *β* are weighting hyperparameters, both set to 1.0 through empirical validation to balance pixel-level accuracy and perceptual quality.

$L_{MSE}=\frac{1}{N}\sum_{i=1}^{N}{\left\|Y_{t}^{\left(i\right)}-{\hat{Y}}_{t}^{\left(i\right)}\right\|}_{2}^{2}$ calculates the pixel-wise mean squared error between the reconstructed image ${\hat{Y}}_{t}$ and the target image $Y_{t}$. This component aims to ensure the reconstruction aligns with the real image in spectral-radiometric properties and forms the foundation for model optimization.

$L_{SSIM}=1-SSIM(Y_{t},{\hat{Y}}_{t})$, where the SSIM index assesses the perceptual quality by comparing the luminance, contrast, and structure of the two images. This component focuses on preserving structural information and local textural characteristics, which is crucial for the visual discriminability of cultural heritage features (e.g., outlines and textures of ancient structures).

This composite design ensures that the model not only pursues pixel-level accuracy but also effectively maintains the structural fidelity and natural appearance of the reconstructed image from a human visual perception. It is particularly suited for cultural heritage remote sensing applications that demand extremely high detail and texture preservation.

#### 2.3.2. Training Strategy and Optimization Details

We designed a focused, end-to-end training pipeline for the proposed complex multi-branch model. Although the model internally contains several sub-networks (e.g., for temporal change learning), the supervisory signal for the entire training process comes solely from the difference between the final reconstruction output ${\hat{Y}}_{t}$ and the real high-resolution image $Y_{t}$ of the target year. This design forces the model to autonomously learn to extract and integrate the most discriminative features and temporal change cues from all input information (including the multi-resolution images from years t − 1, t, and t + 1) to optimally accomplish the reconstruction task for year t, thereby achieving synergistic optimization among the model’s internal components.

Specific optimization and training details are as follows:

Optimizer: The Adam optimizer is used with its default parameters for momentum and decay rates. The initial learning rate is set to 1 × 10^−4^.

Batch Size: The training batch size is set to 25 based on GPU memory capacity.

Dynamic Learning Rate Adjustment: To optimize training dynamics and facilitate convergence to a better local minimum, we implement the ReduceLROnPlateau strategy. Specifically, if the validation loss does not decrease for 3 consecutive epochs, the learning rate is halved. The lower bound for the learning rate is set to 1 × 10^−6^ to prevent training stagnation due to an excessively small learning rate.

Early Stopping: To prevent overfitting and improve training efficiency, we employ an early stopping strategy. If the validation loss shows no improvement over 10 consecutive epochs, the training process is terminated early, and the model checkpoint with the lowest validation loss is restored as the final model.

Through the aforementioned carefully designed training strategy, we ensure not only the stability and efficiency of the training process but also effectively enhance the model’s generalization capability, ensuring that the learned mapping relationship performs well on unseen data.

### 2.4. Experimental Setup and Evaluation Protocol

To systematically and rigorously validate the effectiveness of the proposed temporal change-aware super-resolution model in reconstructing cultural heritage site imagery, we designed the following experimental plan.

#### 2.4.1. Compared Models

We selected representative single-image super-resolution models as baselines to evaluate the performance of our method under the same data conditions:

DRCN: This method employs a Deeply Recursive Convolutional Network, which builds a deep network by recursively applying the same convolutional layer at different depths through parameter sharing. This enhances the model’s receptive field and feature extraction capability without significantly increasing parameters [51].

EDSR: This method constructs a more concise and efficient deep residual architecture by removing redundant modules like batch normalization layers from the original residual network and introducing a residual scaling mechanism. It has demonstrated exceptional performance on multiple super-resolution benchmarks.

Furthermore, to rigorously decompose and verify the contributions of different information sources in our architecture, we carefully designed the following control models:

MultiBranch_Net-LR3: This is an ablation variant of our full method, where the key Temporal Change Learning Branch is removed. It retains only the path for fusing low-resolution information from years t − 1, t, and t + 1. This comparison allows us to directly quantify the contribution of high-resolution temporal change cues to improving reconstruction quality.

MultiBranch_Net-SLR: This is a stricter ablation variant where both the Temporal Change Learning Branch and the multi-temporal low-resolution inputs are removed. It takes only the low-resolution image of the target year t as input, serving to evaluate the baseline capability of our core network backbone when utilizing single-temporal information alone.

SimpleAverage_Baseline: This is a straightforward, non-learning baseline. It generates the reconstruction for the target year t by computing the pixel-wise arithmetic mean of the geometrically registered high-resolution images from the adjacent years t − 1 and t + 1. This model tests the simplest possible use of multi-temporal information without utilizing the low-resolution image of the target year, providing a crucial benchmark to justify the necessity of our cross-resolution fusion design.

By comparing with these models, we can systematically isolate and evaluate the benefits derived from: (a) our network architecture versus established SR models, (b) multi-temporal low-resolution context, (c) high-resolution temporal change priors, and (d) the fusion of target-year observation with adjacent-year information.

#### 2.4.2. Evaluation Metrics

The experiment employs three widely recognized objective image quality assessment metrics to quantitatively evaluate the reconstruction results from different perspectives:

Peak Signal-to-Noise Ratio (PSNR): Measures the pixel-level error between the reconstructed and reference images. A higher value indicates greater pixel accuracy in the reconstruction.

Structural Similarity Index Measure (SSIM): Assesses the similarity in structural information between two images from the perspective of human visual perception. A value closer to 1 indicates better structural fidelity in the reconstructed image.

Mean Squared Error (MSE): Directly computes the average squared difference between pixels. A lower value indicates smaller reconstruction error.

All experiments are conducted on the same training, validation, and test sets to ensure a fair comparison.

## 3. Results

### 3.1. Quantitative Analysis

#### 3.1.1. Training Results

By employing the aforementioned training strategy and experimental setup, we obtained the training results for the different models. It is important to note that the SimpleAverage_Baseline, being a parameter-free, non-learning method, does not undergo a traditional “training” process and thus is not included in this training accuracy comparison. Its performance will be evaluated directly on the test set. The quantitative evaluation results of each model on the training set are summarized in Table 1.

As shown in Table 1, our proposed MultiBranch_Net model demonstrates better performance across three metrics—PSNR, SSIM, and MSE—among the compared baseline methods. This result indicates that our proposed multi-branch architecture, including its core temporal change-aware module, possesses strong feature learning and representation capabilities, enabling it to effectively capture deep features from the training data for high-precision image reconstruction.

An interesting observation is that while MultiBranch_Net-LR3 outperforms the traditional single-image methods DRCN and EDSR, it closely matches the accuracy of MultiBranch_Net-SLR on the training set. This result appears counter-intuitive and likely reflects the training dynamics and task complexity. The MultiBranch_Net-SLR model, with only a single input, faces a simpler mapping problem, which may allow it to converge to a very good local optimum on the training data. In contrast, the MultiBranch_Net-LR3 model must learn to integrate information from three low-resolution images across time, a more complex task that might require more training iterations or a different optimization strategy to fully exploit its potential on the training set. 

It is noteworthy that the MultiBranch_Net-LR3 (the ablated version of our model), while performing better on the training set than the traditional single-image methods EDSR and DRCN, shows a clear performance gap compared to the full MultiBranch_Net. This observation preliminarily confirms the positive role of explicitly modeling temporal change information in enhancing the model’s fitting capability. However, superior performance on the training set is only one aspect of model validity; whether the model has truly learned generalizable patterns, rather than overfitting the training data, must be ultimately verified by its performance on an independent test set.

#### 3.1.2. Test Set Performance

To comprehensively evaluate the generalization capability of the models, we conducted a quantitative assessment of all models on the test set, which was not involved in the training process. The results are detailed in Table 2.

Analyzing Table 2 leads to the following key conclusions:

Overall Leading Performance of Our Model: Our MultiBranch_Net maintains its excellent performance on the test set, achieving a PSNR of 29.54 dB, an SSIM of 0.8413, and a low MSE of 0.0013, ranking first among all compared models. This fully demonstrates that the model not only exhibits strong fitting capability on the training set but also possesses excellent generalization ability, enabling it to effectively apply the learned reconstruction priors to new, unseen data.

Critical Insights from Ablation Models on Generalization: The test set performance reveals a decisive shift in the ranking of the ablated models compared to the training set. Most notably, MultiBranch_Net-LR3 performs significantly worse than MultiBranch_Net-SLR. This is a critical finding: while the MultiBranch_Net-LR3 architecture can leverage multi-temporal low-resolution (LR) context to fit the training data reasonably well (as seen in Table 1), it fails to generalize this knowledge effectively to unseen data. This suggests that low-resolution multi-temporal context alone, without the guiding prior of high-resolution temporal changes, provides an insufficient and potentially noisy signal for robust super-resolution on new scenes. In contrast, MultiBranch_Net-SLR, which focuses on super-resolving a single image, demonstrates more stable generalization. This stark contrast underscores that the mere addition of multi-temporal LR data is not beneficial for generalization; its value is unlocked only when effectively fused with high-resolution temporal semantics, as done in our full model.

Core Demonstration of Temporal Information Value—Comparison with the Ablation Model: The comparison with the ablation model MultiBranch_Net-LR3 is most instructive. On the test set, MultiBranch_Net achieves a remarkable improvement of over 5.26 dB in PSNR compared to MultiBranch_Net-LR3, while SSIM also increases from 0.8106 to 0.8413. Given that the primary difference between the two models is the inclusion of the temporal change learning branch, this substantial performance gap strongly confirms that explicitly learning temporal change patterns from high-resolution images of adjacent years is key to enhancing the model’s reconstruction capability. This suggests that for scenarios like heritage sites, where land cover changes follow certain physical laws (e.g., vegetation growth cycles, anthropogenic restoration projects), incorporating temporal contextual information provides crucial constraints and enriching information for reconstructing the image state of the target year.

Advantage Over Single-Image Methods and Simple Baselines: Compared to the representative single-image super-resolution model EDSR, our model achieves an improvement of nearly 2 dB in PSNR, and the SSIM increases from 0.8082 to 0.8413. Furthermore, the SimpleAverage_Baseline performs the worst among all learning-based methods, quantitatively proving that a naive fusion of adjacent-year high-resolution data is entirely inadequate for reconstructing the target year. This starkly justifies the necessity of our design that fuses the target year’s low-resolution observation with learned temporal priors.

In summary, the quantitative experimental results consistently validate, from various perspectives, the effectiveness and superiority of the proposed temporal change-aware multi-branch super-resolution model. The systematic ablation study, particularly on the test set, provides compelling evidence: the synergy between the target year’s LR observation and the high-resolution temporal change prior is crucial. Neither multi-temporal LR context alone nor a simplistic combination of multi-temporal HR data can achieve generalizable, high-fidelity reconstruction. Our model successfully integrates these elements, providing a new pathway for addressing the “temporal data gap” problem—generating reconstructed data that integrates high spatial detail with temporal consistency.

### 3.2. Qualitative Analysis

Objective quantitative metrics provide an essential benchmark for model performance. However, in remote sensing image analysis, particularly for fine-grained monitoring of cultural heritage sites, the visual fidelity of reconstruction results, the ability to restore detailed features, and the plausibility of spatial structures are equally critical.

#### 3.2.1. Overall Visual Fidelity and Residual Analysis

**Figure 3:**
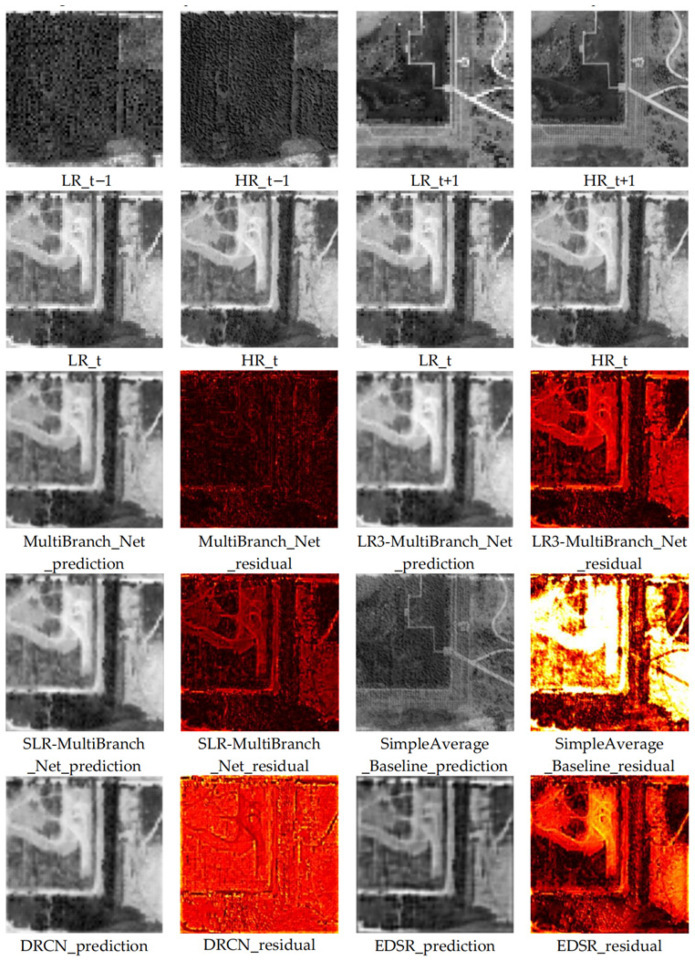
Visual Comparison of Reconstruction Results from Different Models for Representative Area 1.

**Figure 4:**
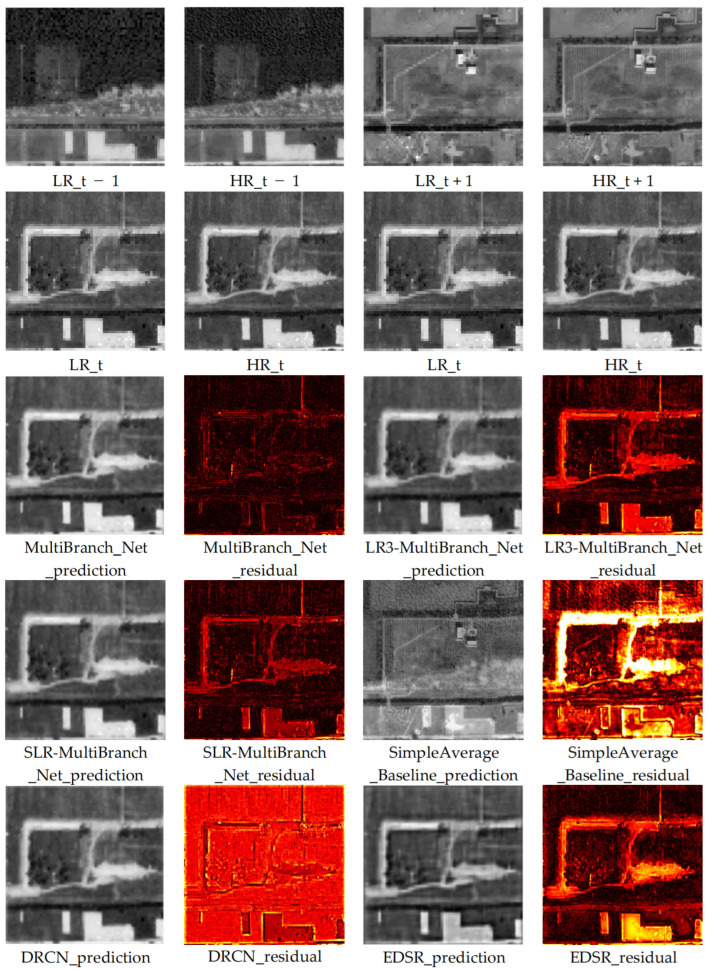
Visual Comparison of Reconstruction Results from Different Models for Representative Area 2.

For a comprehensive qualitative assessment, we generated the reconstruction results of each compared model on the test set for the 2018 low-resolution image and computed their corresponding residual maps against the real high-resolution reference image. This aims to visually reveal the performance differences between methods. Comparison results for typical areas are shown in Figure 3 and Figure 4.

Through detailed visual analysis of the reconstruction results and residual maps, we derive the following observations and conclusions:

Severely Deficient Reconstruction of the Non-Learning Baseline: The SimpleAverage_Baseline produces results with severe errors and is visually the least plausible. This is clearly evident in the residual maps, which show intense, structured errors across entire objects, confirming that a naive arithmetic mean of multi-temporal high-resolution images is incapable of accurate reconstruction for a missing year.

Limitations of Single-Image Models: The reconstruction results of single-image super-resolution models like DRCN and EDSR, while generally enhancing image resolution, exhibit shortcomings in representing key heritage features. Specifically, the reconstructed building edges show blurring and smoothing effects, and the overall images tend to appear oversmoothed and fuzzy. This is clearly evidenced in the residual maps: the residual signals for these models are stronger at object edges and complex texture areas, indicating that a substantial amount of high-frequency structural and detailed information failed to be effectively recovered or was even erroneously reconstructed.

Comparative Analysis of Ablation Models: A side-by-side comparison of the two ablation models reveals crucial insights. The MultiBranch_Net-SLR model, utilizing only the target year’s low-resolution image, produces results that are visually sharper and structurally more coherent than those of DRCN and EDSR. In contrast, the MultiBranch_Net-LR3 model, which incorporates multi-temporal low-resolution contexts, shows surprisingly poorer visual quality. Its results exhibit more pronounced blurring and spatial inconsistencies compared to MultiBranch_Net-SLR, and its residual maps indicate larger and more widespread errors. This visual assessment directly corroborates the quantitative findings on the test set: without the guidance of high-resolution temporal change priors, the added multi-temporal low-resolution information can degrade rather than enhance the reconstruction’s generalization to unseen scenes.

Visual Superiority of Our Proposed Model: Our complete temporal change-aware model (MultiBranch_Net) produces the most visually satisfactory and detail-rich reconstruction results. It appears sharper and exhibits smaller deviations from the real image compared to other models. Specifically, spatial structures such as the boundaries of ancient architectural foundations and road lines are sharper, clearer, and more continuous, effectively suppressing the blurring and distortion artifacts common in other models. The residual map derived from comparison with the real image shows that the residuals from our model are significantly weaker in both overall intensity and spatial distribution than other compared models. This demonstrates that our reconstruction results are closer to the real image in both pixel content and structural information. Crucially, this superior performance is not merely additive but synergistic; it arises specifically from the effective fusion of the target year’s low-resolution observation with the explicitly learned high-resolution temporal prior—a combination uniquely leveraged by our full model and absent in all ablated or baseline methods.

This pronounced visual advantage fundamentally stems from our model’s ability to go beyond relying solely on information from a single image. Through the temporal change learning branch, it gains a deep understanding of the heritage landscape change patterns recorded in the high-resolution imagery from year t − 1 to t + 1. This explicit modeling of “change” enables the model to more intelligently infer the plausible state of the target year (t) within its continuous evolution. Consequently, the generated results are not only more accurate in spatial detail but also more credible in terms of temporal evolution logic. For domain experts relying on high-resolution imagery for heritage site health assessment, damage identification, and change interpretation, such high-quality reconstructed data—capable of restoring authentic details and avoiding the introduction of spurious information—holds crucial practical application value.

#### 3.2.2. Detailed Comparison

To further evaluate the detailed reconstruction capability of each super-resolution model on key elements for cultural heritage monitoring, we selected two representative types of typical features—roads and buildings—for an in-depth detailed comparison. These two feature types are not only important components of the spatial structure of heritage sites, but their clear geometric contours and regular texture patterns are also highly sensitive to the performance of reconstruction algorithms. The comparison results clearly demonstrate significant differences in how well different models restore feature details.

**Figure 5:**
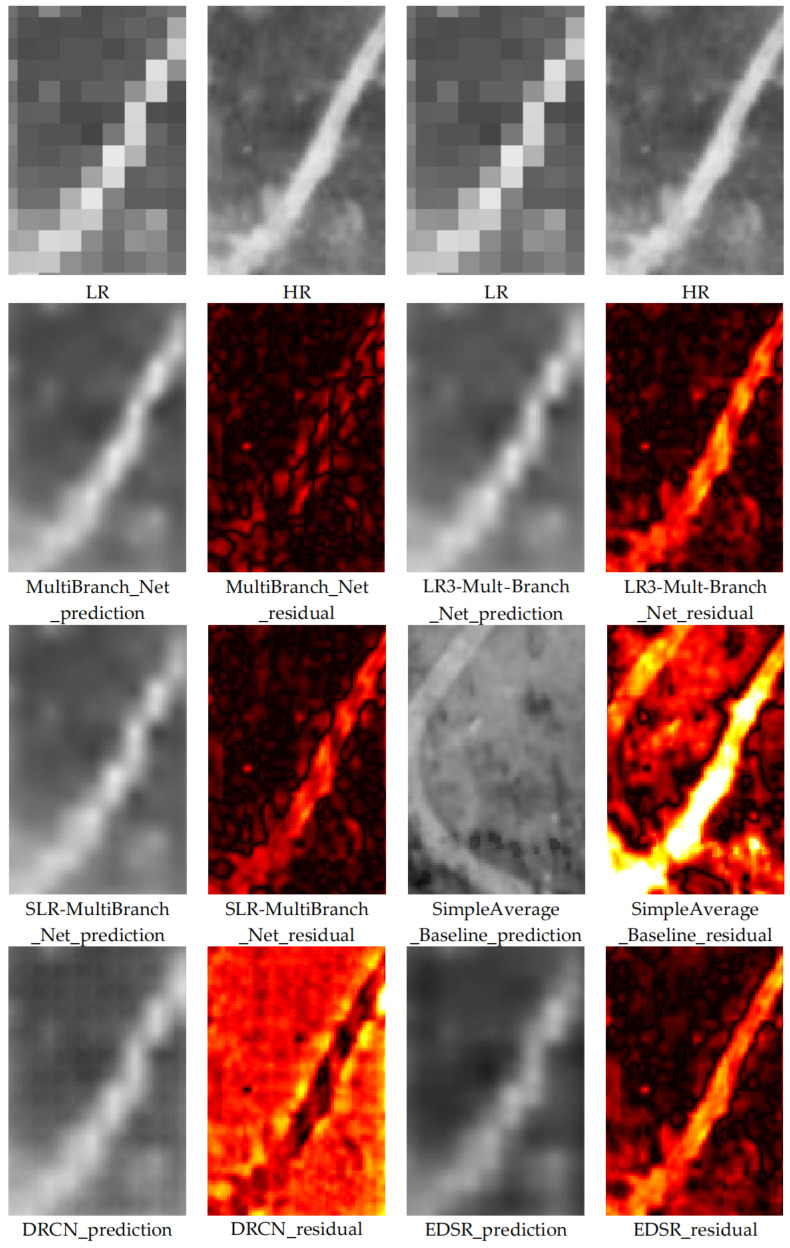
Detailed Visual Comparison of Reconstruction Results from Different Models in Region 1.

**Figure 6:**
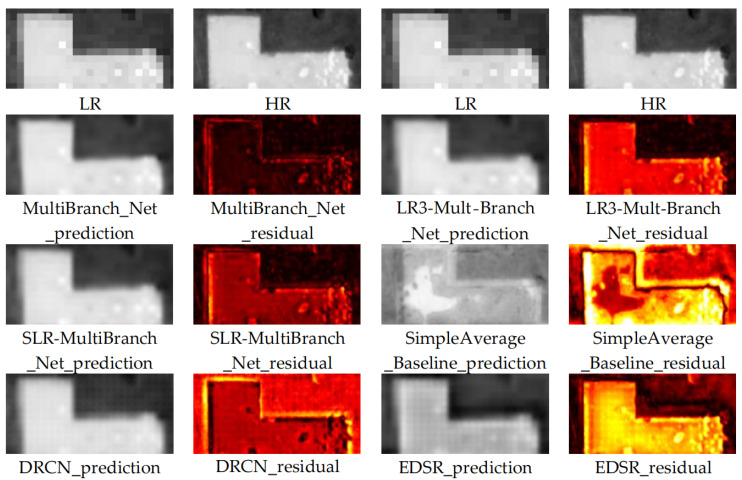
Detailed Visual Comparison of Reconstruction Results from Different Models in Region 2.

Analysis of Figure 5 and Figure 6 reveals the following key observations regarding model performance on these critical features:

Roads: As the skeleton of the heritage site layout, the continuity and smoothness of their linear features are important indicators for evaluating reconstruction quality. Visual comparison (Figure 5) shows that the SimpleAverage_Baseline performs the worst, producing road features with severe errors that are geometrically implausible, essentially an average of the adjacent years’ layouts. In the reconstruction results of single-image models (e.g., EDSR, DRCN), road edges generally exhibit jagged irregularities and discontinuities, failing to effectively restore the desired clear linear characteristics. In contrast, the MultiBranch_Net model produces smoother and more continuous road contours. Its results effectively suppress jagged noise, presenting sharp and natural road edges. This is directly corroborated by the residual maps: our model produces weaker and more sparsely distributed residual signals in the road areas, proving that the restored road pixel values highly align with the true spatial structure. We posit that this advantage stems from the landscape structural priors learned by the model from the temporal context—namely, that the linear characteristics of main roads typically remain stable over short time scales—thus guiding the model to generate results with greater geometric plausibility.

Buildings: The integrity of contours and clarity of edges for structures (e.g., palace wall foundations, rammed earth platforms) are crucial for archaeological interpretation and preservation status assessment. Analysis (Figure 6) reveals that compared models, when reconstructing the regular right-angled edges of buildings, tend to introduce varying degrees of edge blurring and troublesome checkerboard artifacts. These noises not only degrade visual quality but can also severely interfere with subsequent automated contour extraction and change detection.

In contrast, the building edges reconstructed by MultiBranch_Net exhibit superior sharpness and geometric fidelity. The model successfully restores sharp corners and straight boundaries of buildings while significantly eliminating checkerboard artifacts. This indicates that the temporal change-aware branch and feature fusion mechanism in our model can more effectively leverage high-frequency structural cues from the multi-temporal information, working in concert with the low-frequency content from the target frame to jointly constrain the generation process, thereby producing outputs that are closer to the real high-resolution image both visually and metrically.

The detailed analysis of these two typical feature types—roads and buildings—powerfully confirms the advantage of our proposed model in enhancing the practical value of super-resolution reconstruction. It also unequivocally demonstrates that simplistic, non-learning fusion strategies are inadequate for the task. Our model strives not only for pixel-statistical similarity but is also committed to restoring the precise geometric structures and spatial details of key historical remnants within the heritage landscape. This capability makes the reconstructed imagery better suited for professional monitoring and interpretation needs. For instance, clear building edges facilitate accurate contour vectorization and deformation analysis, while smooth, continuous roads aid in studying the evolution of the macroscopic layout. Therefore, our method lays a reliable foundation for generating high-quality data that can directly serve higher-level heritage site analysis applications.

## 4. Discussion

### 4.1. Efficacy and Quantitative Assessment of the Proposed Model

This study introduces a novel temporal change-aware super-resolution reconstruction model to address the core bottleneck of “temporal data gaps” in the dynamic monitoring of World Cultural Heritage sites. Through a meticulously designed multi-branch architecture capable of explicitly learning the temporal evolution patterns of heritage landscapes, our model successfully leverages temporal contextual information from adjacent years (t − 1 and t + 1) as a key driver to enhance the reconstruction quality and ensure the temporal consistency of the target year (t) imagery.

Systematic experimental results on the WYP site demonstrate the significant advantages of our model. Quantitatively, it surpassed advanced single-image models (DRCN, EDSR) and the ablation model (MultiBranch_Net-LR3) across objective metrics: achieving the highest PSNR and SSIM, alongside the lowest MSE. Notably, the superior SSIM scores specifically indicate better preservation of structural fidelity and local textural details—attributes critical for discerning subtle archaeological features. The consistent enhancement in PSNR, particularly at the 8x upscaling factor, confirms the model’s effectiveness in spatial resolution enhancement and fine detail recovery. Qualitatively, its reconstruction results exhibited superior visual fidelity in both road edge sharpness and building contour integrity. Crucially, by comparing with the ablation model, we have robustly confirmed that explicitly modeling temporal change information is central to achieving this performance gain, rather than merely the introduction of multi-temporal data.

To place our work in a broader context, we note that while recent multi-temporal super-resolution works (e.g., spatio-temporal sub-pixel networks [46], recurrent encoder–decoder frameworks [47]) have shown promise in video domains, their direct applicability to remote sensing heritage monitoring is limited. These methods are typically optimized for short-term, high-frame-rate sequences with consistent motion patterns. In contrast, our model is explicitly architected for the distinct challenges of heritage remote sensing: long intervals (annual acquisitions), non-uniform change rates (e.g., slow weathering vs. seasonal vegetation cycles), and the need to prioritize the reconstruction of static or semi-static cultural features. This targeted design accounts for our model’s demonstrated superiority over generic single-image benchmarks in this domain-specific task.

### 4.2. Practical Implications, Limitations, and Generalizability

The core value of this work lies in elevating the role of deep learning-based super-resolution technology from a mere “image sharpening tool” to a “data enhancement and restoration engine” that serves deep temporal understanding and analysis of heritage sites. Compared to traditional methods, the outputs of our model are not only superior in pixel accuracy but, more importantly, the generated high-resolution imagery can more authentically reflect the historical state the target year should occupy within the continuous evolution of the heritage site. This ensures the logical plausibility of the reconstruction within the time series. This unique “temporal consistency” capability endows the reconstructed data with profound application potential. For practical monitoring workflows, this method can be integrated into documentation pipelines to generate plausible, high-resolution imagery for years with poor-quality or missing acquisitions. This directly enhances the continuity of digital archives, providing conservators with a more complete spatiotemporal dataset for condition assessment, change detection, and the planning of preservation interventions. It provides a more reliable data foundation for tasks like erosion monitoring, ultimately offering high-quality, spatiotemporally plausible image estimates for key years suffering from “monitoring gaps.”

However, the performance of the current framework is subject to several limitations that define its current scope and future directions:

Sensitivity to Data Quality and Alignment: The model’s performance is contingent upon high-quality input data. It is sensitive to significant misalignment (despite our sub-pixel registration), strong sensor noise variability, and drastic changes in lighting/atmospheric conditions across acquisitions. These factors can degrade the learned temporal correlations. Future improvements could integrate learning-based feature alignment strategies or temporal attention mechanisms to dynamically weight reliable features and suppress noise across time steps.

Analysis of Challenging Reconstruction Patterns: While our quantitative results confirm the model’s overall effectiveness, its performance is inherently tied to the nature of temporal changes in the scene. We observe that the method excels at reconstructing stable or regularly changing features (e.g., the overall layout of archaeological remains, large building footprints, and areas with cyclical vegetation). This is expected as such features provide strong and consistent temporal constraints for the model. Conversely, the reconstruction of fine local details or areas subject to irregular, abrupt changes (such as those caused by human activity between acquisitions) is inherently more challenging and may exhibit lower fidelity. This pattern underscores the model’s fundamental design principle and strength: its reliance on learning and leveraging coherent, predictable temporal evolution logic to inform the reconstruction.

Computational Considerations: In terms of practical feasibility, the proposed model exhibits a moderate computational cost characteristic of contemporary deep learning-based super-resolution architectures. The memory usage scales with the number and size of the input multi-temporal patches. While the training process is resource-intensive, requiring several hours to a day on a modern GPU, the inference (prediction) for a typical site-scale area is computationally efficient, completing within minutes. While the model is viable for operational processing at the scale of individual heritage sites, its application to very large landscapes or real-time monitoring would require future enhancements in computational efficiency, such as model simplification, tiling, or the development of a distilled lightweight version.

Generalization Across Heritage Types: The current validation is focused on a large earthen archaeological site (WYP). Generalizing across diverse heritage types (e.g., stone monuments, wooden buildings, cave temples) and scales may require adaptation. Different materials and deterioration processes (e.g., salt weathering on stone vs. erosion on earthworks) exhibit distinct temporal signatures in imagery. Future work must systematically validate the method’s transferability and potentially adapt the temporal change learning branch or incorporate material-specific prior knowledge to enhance its applicability to cultural heritage.

## 5. Conclusions

In conclusion, this study proposes and validates a temporal change-aware super-resolution model designed to mitigate the problem of temporal data gaps in cultural heritage monitoring. The model architecture is explicitly tailored to learn from the inherent evolution patterns in multi-temporal remote sensing imagery.

The results indicate that the proposed model offers improved reconstruction performance over conventional single-image super-resolution baselines, while also yielding outputs with enhanced temporal consistency. This shift from producing only a sharper image to generating a temporally plausible data product adds significant value to digital heritage archives and strengthens their utility for longitudinal studies.

The approach shows potential for integration into heritage documentation workflows, particularly in scenarios with well-registered image series and coherent change patterns. Future efforts should aim to improve the model’s robustness to common data imperfections, explore its integration with downstream analytical tasks, and rigorously assess its generalizability across diverse heritage typologies. Further incorporation of domain-specific knowledge regarding heritage decay and seasonal dynamics will be important to better translate this methodology from a theoretical solution into a practical tool for heritage science and sustainable conservation.

## Figures and Tables

**Table 1:** Comparative Training Accuracy of Different Models.

Model	PSNR	SSIM	MSE
MultiBranch_Net	28.9775	0.8417	0.0015
MultiBranch_Net-LR3	28.1596	0.8315	0.0017
MultiBranch_Net-SLR	28.2058	0.8302	0.0017
DRCN	23.8621	0.7921	0.0076
EDSR	28.0291	0.8180	0.0018

**Table 2:** Comparative Testing Accuracy of Different Models.

Model	PSNR	SSIM	MSE
MultiBranch_Net	29.5394	0.8413	0.0013
MultiBranch_Net-LR3	24.2766	0.8106	0.0043
MultiBranch_Net-SLR	27.8612	0.8241	0.0018
SimpleAverage_Baseline	18.0618	0.5649	0.0186
DRCN	17.9934	0.7659	0.0159
EDSR	27.6192	0.8082	0.0019

## Data Availability

The original contributions presented in this study are included in the article. Further inquiries can be directed to the corresponding author.
